# Serum Amyloid a Promotes Visfatin Expression in Macrophages

**DOI:** 10.1155/2016/4819327

**Published:** 2016-02-24

**Authors:** Shixun Wang, Xincai Zhang, Lei Tan

**Affiliations:** Department of Cardiology II, Weifang People's Hospital, Weifang 261041, China

## Abstract

Visfatin has been reported to exert an important role in the development of atherosclerosis. However, the mechanism that regulated the expression of Visfatin has not been elucidated yet. This study aimed to investigate the effect of SAA on the regulation of Visfatin, as well as the potential pathway. After RAW264.7 macrophages and primary monocytes were stimulated with SAA, the mRNA and protein expression of Visfatin was detected with real-time PCR and western blot, respectively. The concentration of Visfatin in the supernatant was measured with ELISA. Formyl peptide receptor 2 (FPR2) agonist (WKYMVm) and inhibitor (WRW^4^), extracellular signal-regulated kinase 1/2 (ERK1/2) inhibitor (PD98059), and peroxisome proliferator-activated receptor-*γ* (PPAR-*γ*) agonist (Rosiglitazone) and inhibitor (GW9662) were used to investigate the mechanism of regulation of Visfatin. The results demonstrated that SAA upregulated Visfatin expression in cultured RAW264.7 macrophages and in the primary monocytes. WRW^4^ decreased SAA-induced Visfatin production, while WKYMVm could induce Visfatin expression. PD98059 reduced SAA-induced Visfatin production. What is more, GW9662 inhibited SAA-induced Visfatin production, while Rosiglitazone promoted Visfatin expression. These results demonstrate that SAA upregulates Visfatin expression via a FPR2/ERK1/2/PPAR-*γ* signaling pathway.

## 1. Introduction

Visfatin, also known as nicotinamide phosphoribosyltransferase (Nampt) and pre-B-cell-colony-enhancing factor (PBEF), was firstly found in the visceral fat, especially the perivascular fat near the aorta or coronary artery [1, 2]. Meanwhile, activated monocytes/macrophages are also seen as another important source of Visfatin. Visfatin has been reported to play an important role in a series of biological reactions and exhibit proinflammatory and proangiogenic properties. Visfatin induced the expressions of intercellular cell adhesion molecule-1 (ICAM-1) and vascular cell adhesion molecule-1 (VCAM-1) and thus promoted leukocyte adhesion to endothelial cells [3]. Visfatin showed its proangiogenic effect through inducing monocyte chemotactic protein-1 (MCP-1) and its receptor C-C chemokine receptor-2 (CCR-2) production [4]. Visfatin also showed its ability to induce the expression of IL-6, IL-8 [5], MMP-2, and MMP-9 [6], which are the crucial cytokines that could change the status of the atherosclerotic plaques. Therefore, a positive association of Visfatin with atherosclerosis and coronary artery disease (CAD) has been identified [7]. However, it remains unclear how the expression of Visfatin is regulated. Therefore, it is imperative for us to investigate and clarify the mechanism that underlies Visfatin regulation.

Serum amyloid A (SAA), an acute-phase reactant, is normally present in the blood at a concentration of 0.1 *μ*M but increases up to 1000-fold in response to a variety of stress reactions [8, 9]. SAA has been implicated in a variety of chronic inflammatory diseases, especially atherosclerosis. A series of recent studies have suggested that SAA may play a role not only as a responder or a passive participant, but also as an active perpetrator in the inflammatory process [10]. SAA has been reported to promote the chemotaxis, migration, and adhesion of inflammatory cells, especially monocytes/macrophages. SAA has also showed its ability to induce the synthesis and secretion of multiple kinds of inflammatory cytokines, such as TNF-*α*, IL-1*β*, MCP-1, and Lp-PLA_2_ [11–13]. However, there is still no data currently regarding the functions of SAA on the expression of Visfatin. Therefore, based on the results above, we made an assumption that the expression of Visfatin can be modulated by SAA and designed the experiments to validate the hypothesis.

## 2. Materials and Methods

### 2.1. Reagents

Recombinant human apo-SAA1 was purchased from PeproTech (Rocky Hill, NJ) and Sigma-Aldrich (St. Louis, MO). Rabbit monoclonal anti-Visfatin was purchased from Abcam (Cambridge, UK). Mouse polyclonal antibody for peroxisome proliferator-activated receptor-*γ* (PPAR-*γ*) was purchased from Bioss (Beijing, China). Visfatin Elisa Kit was also purchased from Bioss (Beijing, China). Rabbit polyclonal antibodies for total-ERK1/2 and p-ERK1/2 were both purchased from Cell Signaling Technology Inc. (Danvers, MA). Mouse anti-GAPDH was purchased from Bioworld Technology (Minneapolis, USA). FPR2 siRNA for both mice and humans was purchased from Santa Cruz Biotechnology (Dallas, TX). H2N-WRWWWW-CONH2 (WRW^4^), a FPR2 antagonist, was purchased from Tocris Bioscience (Ellisville, Missouri). WKYMVm trifluoroacetate salt, a peptide agonist of formyl peptide receptors, was purchased from Sigma-Aldrich (St. Louis, MO). PD98059 (a selective ERK1/2 inhibitor) was purchased from Beyotime Institute of Biotechnology (Jiangsu, China). PPAR-*γ* agonist Rosiglitazone and PPAR-*γ* antagonist GW9662 were purchased from Sigma-Aldrich (St. Louis, MO).

### 2.2. Cell Culture

RAW264.7 cells were obtained from American Type Culture Collection (Manassas, VA, USA). RAW264.7 cells were cultured in DMEM (Hycolone) supplemented with 5% fetal bovine serum (Hycolone) at 37°C in a humidified atmosphere of 5% CO_2_. Human peripheral blood mononuclear cells (PBMCs) were isolated from anticoagulated peripheral blood by using Histopaque-1077 (Sigma-Aldrich). Then, the cells were placed in 0.5 mL of RPMI 1640 supplemented with 10% fetal bovine serum (Hycolone) in 12-well plates (5 × 10^6^ cells/well). After 2 h, the nonadherent cells were removed and most of the adherent cells were monocytes.

### 2.3. Quantitative Real-Time PCR

After being stimulated by SAA or other agents, RAW264.7 cells were harvested for RNA extraction. Total RNA was extracted from cultured RAW264.7 cells with TranZol Up (Trans, China), with its quality and quantity analyzed by spectrophotometry. After 1 *μ*g of the mRNA was reverse-transcribed using the First Strand cDNA Synthesis Kit (Fermentas, UAB), the amplification of the total cDNA was performed with the Light Cycler (Roche, Basel, Switzerland) real-time PCR detection system using the First Start Universal SYBR Green Master (ROX) (Roche, Swiss Confederation) for 40 cycles at 95°C for 10 sec, 60°C for 20 sec, and 72°C for 30 sec. 18S rDNA was chosen as the reference gene. The primer sequences for real-time PCR analyses were as follows: mouse Visfatin, forward primer: 5′-GGCCACAAATTCCAGAGAACAG-3′ and reverse primer: 5′-CCAAATGAGCAGATGCCCCTAT-3′ [14]; and 18S, forward primer: 5′-CTTAGTTGGTGGAGCGATTTG-3′ and reverse primer: 5′-GCTGAACGCCACTTGTCC-3′ [13].

### 2.4. Western Blot

After being stimulated by SAA or other agents, RAW264.7 cells were harvested for protein extraction. The extracted protein concentration was measured using the bicinchoninic acid (BCA) method. The proteins were separated in 10% SDS-polyacrylamide gel and blotted onto polyvinylidene difluoride membranes (Pierce), which were then blocked by 5% nonfat dry milk in TBS-T [20 mmol/L Tris-HCl (pH 8.0), 8 g/L NaCl, and 0.1% Tween 20] for 2 h at room temperature. After that, the membranes were incubated with specific primary antibodies at 4°C overnight. The membranes were probed with horseradish peroxidase (HRP) conjugated secondary Ab for an additional two hours at room temperature. Antigen-antibody complexes were detected with an electrochemiluminescence (ECL) detection system (Millipore, USA). The results were analyzed with Quantity One (Bio-Rad, USA). The expression level of sample was indicated as a ratio of sample to GAPDH.

### 2.5. Enzyme-Linked Immunosorbent Assay

RAW264.7 macrophages and primary monocytes were cultured in 96-well plates. After stimulation with SAA or other reagents, cell-free supernatants were collected, centrifuged, and assayed for Visfatin by ELISA (RayBiotech) according to the manufacturer's instructions.

### 2.6. Transfection of siRNA

RAW264.7 macrophages and primary monocytes were seeded at 5 × 10^6^ per well in 12-well plates and allowed to adhere overnight in serum-containing DMEM or RPMI 1640. FPR2 siRNA (0.02 nmol) was mixed with 3 *μ*L Lipofectamine 2000 (Invitrogen, USA) and 100 *μ*L OPTI-MEM transfection medium (Invitrogen) and then cultured with the cells for 6 h. After that, the supernatant was removed and fresh medium was added for further experiments.

### 2.7. Data Analysis

Results were expressed as means ± SEM. Statistical comparisons used one-way ANOVA. *P* < 0.05 was considered statistically significant.

## 3. Results

### 3.1. SAA Upregulates Visfatin Expression in RAW264.7 Cells at Both Protein and mRNA Levels

**Figure 1:**
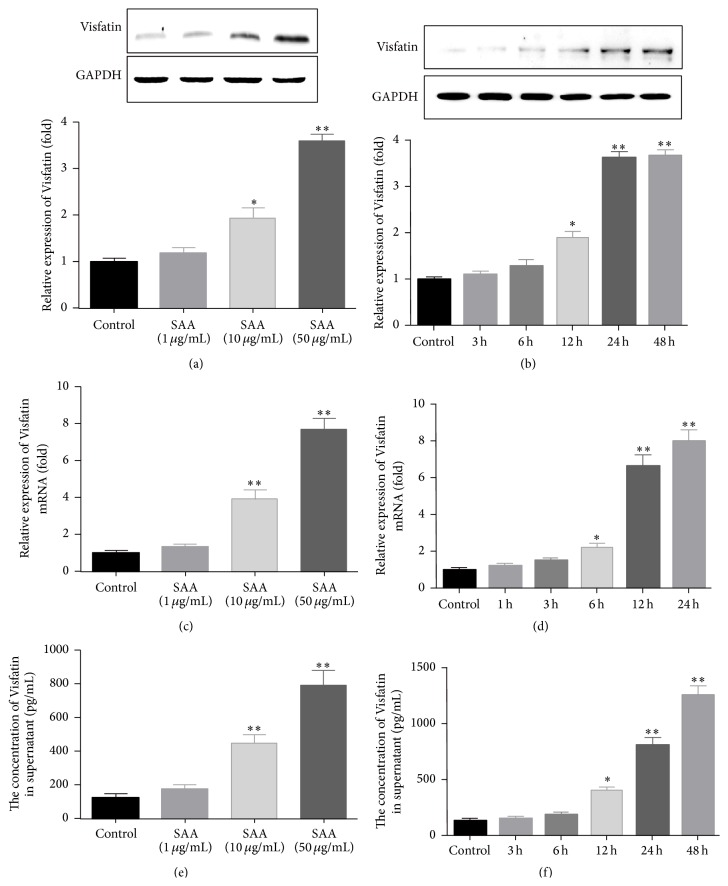
SAA upregulates Visfatin expression in cultured RAW264.7 cells. (a, c, e) RAW264.7 cells were stimulated with different concentrations of SAA (0, 1, 10, and 50 *μ*g/mL) for 24 h. The expression of Visfatin in the cells was detected with western blot and real-time PCR, respectively, at protein and mRNA levels, while ELISA was used to detect the concentration of Visfatin in the supernatants. (b, d, f) RAW264.7 cells were stimulated with 50 *μ*g/mL SAA for different times (0, 3, 6, 12, 24, and 48 h). The expression of Visfatin was detected with western blot and real-time PCR, respectively, at protein and mRNA levels, while ELISA was used to detect the concentration of Visfatin in the supernatants. ^*∗*^ *P* < 0.05 versus control group; ^*∗∗*^ *P* < 0.01 versus control group. Data shown are means ± SEM from three independent experiments in duplicate.

To investigate the effect of SAA on Visfatin expression in RAW264.7 cells, cells were cultured in 6-well dishes and stimulated with different concentrations of SAA (0, 1, 10, and 50 *μ*g/mL) for 24 h. After collecting the cells, we extracted the proteins and detected the expression of Visfatin with western blot. The results demonstrated that, after stimulation with SAA, the relative expression of Visfatin was not significantly increased when the concentration of SAA was 1 *μ*g/mL, but 10 and 50 *μ*g/mL of SAA significantly increased Visfatin expression compared with control group (Figure 1(a)). Then, 50 *μ*g/mL of SAA was used to stimulate the cells for different time (0, 3, 6, 12, 24, and 48 h), and the results showed that the stimulation did not significantly enhanced the Visfatin expression until the stimulation time reached 12 h (Figure 1(b)). We also found that when we prolonged the stimulation time to 24 h and 48 h, SAA reached its peak activity.

In order to further confirm the effect of SAA on Visfatin production, the cells were cultured and stimulated again, and the mRNA of Visfatin was detected with real-time PCR. After stimulation with SAA at the concentrations (0, 1, 10, and 50 *μ*g/mL) for 24 h, we found that the relative expression of Visfatin mRNA was not significantly increased compared with control group in the 1 *μ*g/mL group, but when the concentration increased to 10 and 50 *μ*g/mL, the relative expressions of Visfatin mRNA were gradually enhanced and reached significant differences. After that, 50 *μ*g/mL of SAA was again used to stimulate the cells for different time (0, 1, 3, 6, 12, and 24 h), and the results showed that the stimulation effect of SAA on Visfatin mRNA expression was gradually enhanced with the increase of stimulation time (Figure 1(b)), appeared significantly different after 6 h, and reached peak after 24 h.

After that, we collected the supernatants of the cells that were stimulated by SAA of different concentrations and times. The concentrations of Visfatin were detected with ELISA and we also obtained similar results that SAA significantly increased the concentrations of Visfatin in the supernatants (Figures 1(e) and 1(f)).

In addition, to further confirm the effect of SAA, we also purchased recombinant human apo-SAA1 from Sigma-Aldrich (St. Louis, MO). RAW264.7 macrophages were again cultured and stimulated with SAA (50 *μ*g/mL, Sigma, SRP4324) for 24 h. The expression of Visfatin was also significantly elevated (Supplemental Figure 1 in Supplementary Material available online at http://dx.doi.org/10.1155/2016/4819327). As a result, according to the data above, we concluded that SAA induced Visfatin upregulation in concentration- and time-dependent manners.

### 3.2. FPR2 Mediated SAA-Induced Visfatin Production

**Figure 2:**
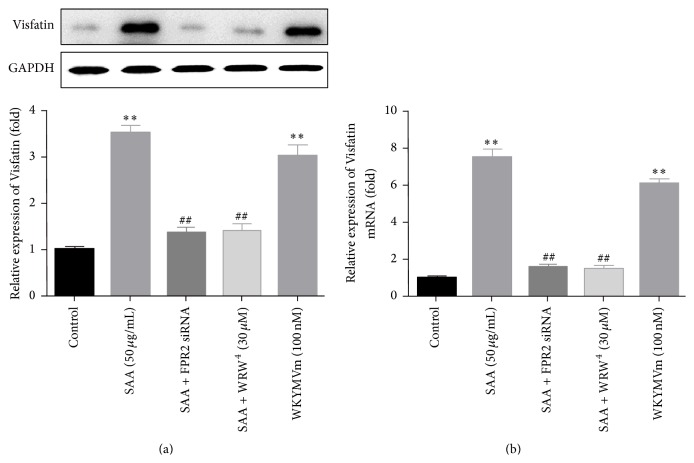
FPR2 mediated SAA-induced Visfatin production in RAW264.7 cells. (a) RAW264.7 cells were pretreated with FPR2 siRNA (20 nM) for 6 h and with WRW^4^ (30 *μ*M) for 1 h before the cells were stimulated with SAA. Then, WKYMVm (100 nM, a FPR2 agonist) was also used to treat the cells for 24 h. The expression of Visfatin was detected with western blot at protein level. (b) Real-time PCR showed a similar result. ^*∗∗*^ *P* < 0.01 versus control group; ^##^ *P* < 0.01 versus SAA treatment group. Data shown are means ± SEM from three independent experiments in duplicate.

FPR2 has been reported to play important roles in multiple biological processes [10, 15, 16], and has been seen as one of the most important receptors of SAA on the membranes. In the present study, in order to clarify the role of FPR2 in the process where SAA induced the expression of Visfatin, we firstly pretreated the RAW264.7 cells with FPR2 siRNA (20 nM) for 6 h and with WRW^4^ (30 *μ*M, the FPR2 inhibitor) for 1 h before the cells were stimulated with SAA. The results (Figure 2(a)) demonstrated that SAA-induced Visfatin production could be blocked by the pretreatment of either FPR2 siRNA or WRW^4^. Then, WKYMVm (100 nM, a FPR2 agonist) was also used to treat the cells for 24 h, and we also found that it evidently promoted Visfatin production compared with control group (Figure 2(a)), just like the effect of SAA. Simultaneously, we also extracted the mRNA and detected the expression of Visfatin at the mRNA level. It showed a similar result in accordance with the protein level changes under the same stimulation depicted above (Figure 2(b)).

### 3.3. SAA-Induced Visfatin Production in Primary Monocytes via FPR2

**Figure 3:**
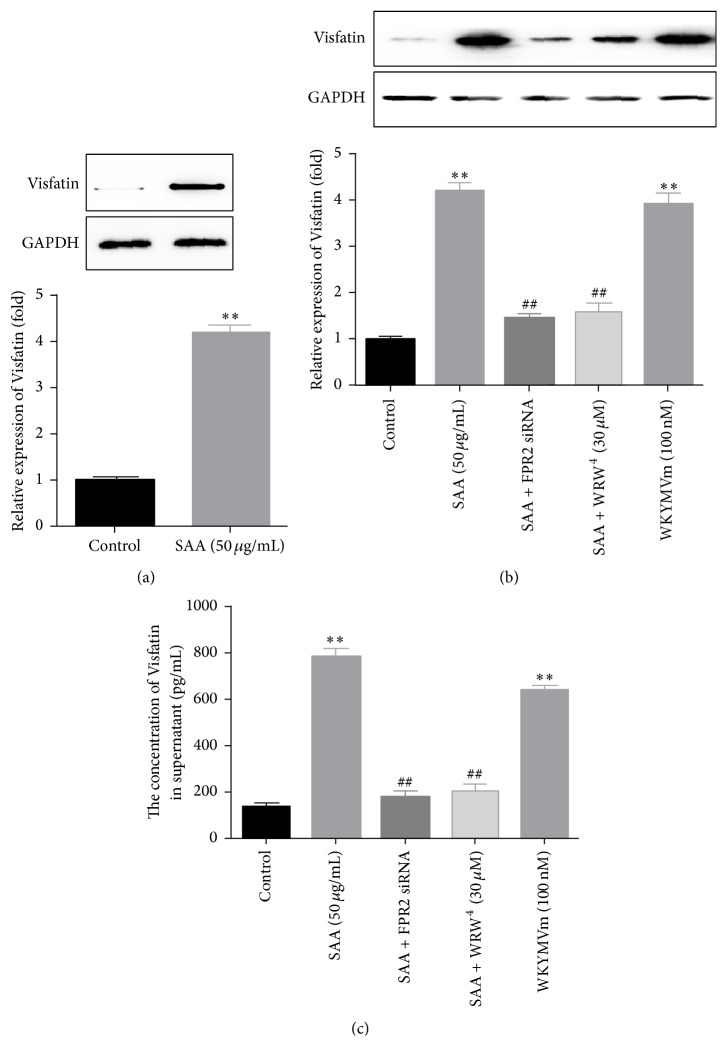
FPR2 mediated SAA-induced Visfatin production in primary monocytes. (a) Primary monocytes were isolated and cultured and then stimulated with SAA (50 *μ*g/mL). The expression of Visfatin was detected with western blot at protein level. (b) Primary monocytes were pretreated with FPR2 siRNA (20 nM) for 6 h and with WRW^4^ (30 *μ*M) for 1 h before the cells were stimulated with SAA. Then, WKYMVm (100 nM, a FPR2 agonist) was also used to treat the cells for 24 h. The expression of Visfatin was detected with western blot at protein level. (c) The concentration of Visfatin in the supernatants was detected with ELISA. ^*∗∗*^ *P* < 0.01 versus control group; ^##^ *P* < 0.01 versus SAA treatment group. Data shown are means ± SEM from three independent experiments in duplicate.

In order to confirm the effect of SAA on the expression of Visfatin in primary monocytes, circulating human monocytes isolated from human whole blood were cultured with SAA (50 *μ*g/mL) for 24 h. The results demonstrated that Visfatin was significantly increased after the stimulation (Figure 3(a)). Then, we pretreated the primary monocytes with FPR2 siRNA (20 nM) for 6 h and with WRW^4^ (30 *μ*M) for 1 h before the cells were stimulated with SAA (50 *μ*g/mL) or treated the primary monocytes with WKYMVm (100 nM). The results (Figure 3(b)) demonstrated that SAA-induced Visfatin production could be blocked by the pretreatment of either FPR2 siRNA or WRW^4^. Meanwhile, WKYMVm could also promote Visfatin production compared with control group, just like the effect of SAA. Then, we also detected the concentration of Visfatin in the supernatants, and we got similar results (Figure 3(c)).

### 3.4. SAA-Induced Visfatin Production Was Mediated by ERK1/2

**Figure 4:**
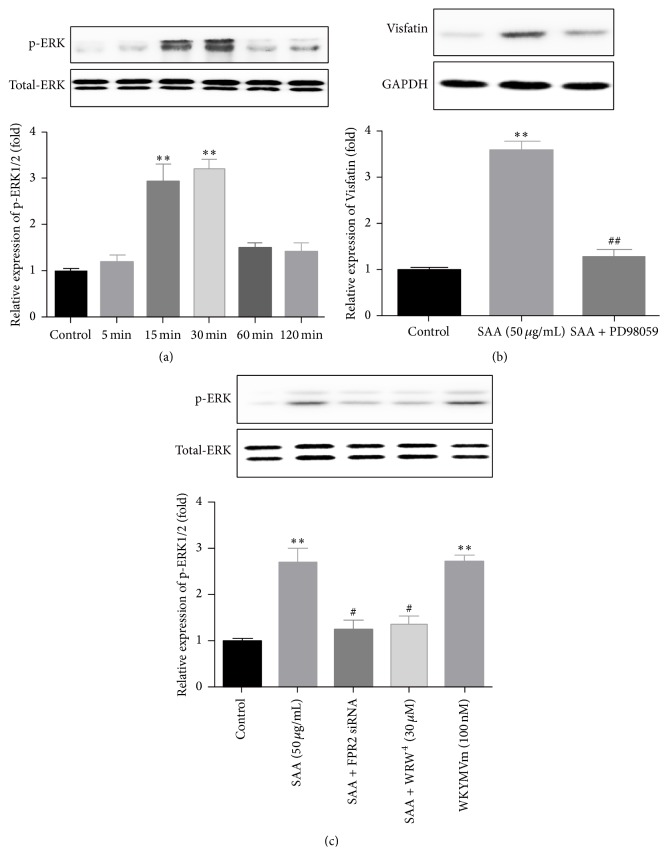
ERK1/2 mediated SAA-induced Visfatin production. (a) RAW264.7 cells were stimulated with 50 *μ*g/mL SAA for different times (0 min, 5 min, 15 min, 30 min, 1 h, and 2 h). The phosphorylation of ERK1/2 was detected with western blot. (b) RAW264.7 cells were preincubated with PD98059 (20 *μ*mol/L) and then treated with 50 *μ*g/mL SAA for 24 hours. The expression of Visfatin was detected with western blot at protein level. (c) After pretreatment with FPR2 siRNA (20 nM, for 6 h) and WRW^4^ (30 *μ*M, 1 h) before SAA (50 *μ*g/mL) for another 24 h, or stimulation of WKYMVm (100 nM) for 24 h, phosphorylation of ERK1/2 was measured with western blot. ^*∗∗*^ *P* < 0.01 versus control group; ^#^ *P* < 0.05 versus SAA treatment group; ^##^ *P* < 0.01 versus SAA treatment group. Data shown are means ± SEM from three independent experiments in duplicate.

It has been reported that ERK1/2 was a downstream signaling molecule of SAA, which played important roles in a series of biological processes that SAA participated in [17, 18]. Thus, in the present study, we want to know whether SAA-induced Visfatin production was mediated by ERK1/2. We firstly assessed the expression of ERK1/2 after stimulating the RAW264.7 cells with SAA (50 *μ*g/mL) for 5, 15, 30, 60, and 120 min. The results demonstrated that the relative expression of p-ERK1/2 was not increased after 5-minute stimulation but was quickly upregulated and reached peak after 15 and 30 min stimulation and then fell back after 60 and 120 min stimulation (Figure 4(a)). Then, the specific inhibitor of ERK1/2, PD98059 (20 *μ*M), was used to block the ERK1/2 pathway, and the inhibitor showed a significant role to abolish the elevated role of SAA (Figure 4(b)), which further confirmed the critical effect of ERK1/2. We further evaluated the effect of FPR2 on the phosphorylation of ERK1/2. When we pretreated the cells with FPR2 siRNA (20 nM) for 6 h and with WRW^4^ (30 *μ*M, the FPR2 inhibitor) for 1 h before the cells were stimulated with SAA for another 30 min, the expression of p-ERK1/2 was obviously decreased (Figure 4(c)). However, when we treated the cells with single WKYMVm (100 nM) for 30 min, the expression of p-ERK1/2 was obviously increased (Figure 4(c)). The results above suggested that SAA-induced Visfatin production was mediated by FPR2 and ERK1/2, and the phosphorylation of ERK was mediated by FPR2.

### 3.5. SAA-Induced Visfatin Production Was Mediated by PPAR-*γ*

**Figure 5:**
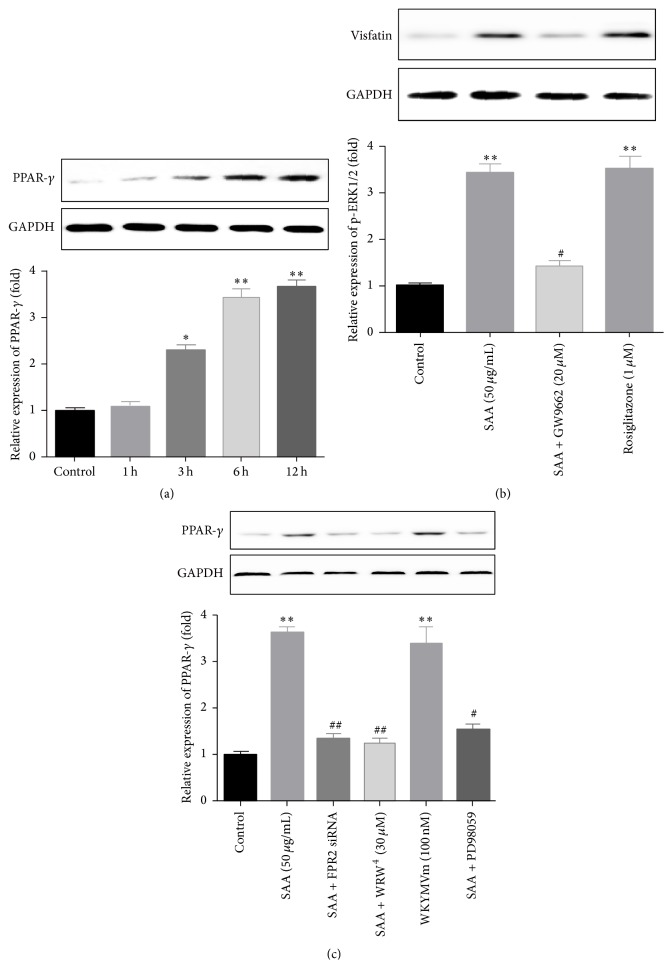
PPAR-*γ* mediated SAA-induced Visfatin production. (a) RAW264.7 cells were stimulated with 50 *μ*g/mL SAA for different times (1 h, 3 h, 6 h, and 12 h). Western blot showed that SAA significantly promoted the expression of PPAR-*γ*. (b) RAW264.7 cells were incubated for 12 hours with SAA alone (50 *μ*g/mL) or combined with GW9662 (20 *μ*M) for pretreatment or with Rosiglitazone alone (1 *μ*M). Western blot showed the expression of changes of Visfatin expression. (c) After pretreatment with FPR2 siRNA (20 nM) for 6 h, WRW^4^ (30 mM) for 1 h, and PD98059 (20 *μ*mol/L) for 2 h before SAA (50 *μ*g/mL) for another 24 h, or stimulation of WKYMVm (100 nM) for 24 h, expression of PPAR-*γ* was measured with western blot. ^*∗*^ *P* < 0.05 versus control group; ^*∗∗*^ *P* < 0.01 versus control group; ^#^ *P* < 0.05 versus SAA treatment group; ^##^ *P* < 0.01 versus SAA treatment group. Data shown are means ± SEM from three independent experiments in duplicate.

As a nuclear transcription factor of SAA and ERK1/2, PPAR has also been reported to play important roles in multiple biological processes together with ERK1/2 [13, 17]. Therefore, we also wanted to determine whether PPAR-*γ* could mediate SAA-induced Visfatin production in our study. The expression of Visfatin was detected after stimulation with SAA for different time (0, 1, 3, 6, and 12 h), and the results showed that the expression of PPAR-*γ* was significantly increased after 3 h of stimulation with SAA and reached the summit after 6–12 h treatment (Figure 5(a)). Then, the specific antagonist and agonist of PPAR-*γ* signaling pathway were used to further clarify the effect of PPAR-*γ* in the reaction. The pretreatment of GW9662 (20 *μ*M) for 2 h before SAA for the other 24 h significantly abolished the effect of SAA on PPAR-*γ* expression, while using Rosiglitazone (1 *μ*M) alone significantly promotes the expression of PPAR-*γ* (Figure 5(b)). We further evaluated the effects of FPR2 and ERK1/2 on the expression of PPAR-*γ*. When we pretreated the cells with FPR2 siRNA (20 nM) for 6 h and with WRW^4^ (30 *μ*M, the FPR2 inhibitor) for 1 h before the cells were stimulated with SAA for another 6 h, the expression of PPAR-*γ* was obviously decreased (Figure 5(c)). However, when we treated the cells with single WKYMVm (100 nM) for 6 h, the expression of p-ERK1/2 was obviously increased (Figure 5(c)). We also pretreated the cells with ERK1/2 pathway inhibitor PD98059 (20 *μ*M) before SAA for another 6 h, and the result showed that the pretreatment also inhibited the expression of PPAR-*γ* (Figure 5(c)). These results supported the notion that the SAA-induced increase of Visfatin expression was dependent on FPR2/ERK1/2/PPAR-*γ* pathway.

## 4. Discussion

Visfatin, another important inflammatory cytokine, has also exhibited its proinflammatory and proangiogenic properties, which has been reported to localize to foam cell macrophages within unstable atherosclerotic lesions and plays a role in plaque destabilization [7]. However, very little data are currently available regarding the generation of Visfatin in vitro or in vivo, so it is essential to investigate the mechanism that regulates the expression of Visfatin. On the other hand, as one of the most important acute-phase proteins, SAA can be significantly increased both systemically in general circulation and locally within impaired tissues in response to inflammatory stimulation. SAA has been widely considered not only as a biomarker of inflammation and atherosclerosis, but also as a proinflammatory mediator that could directly stimulate the production of series of cytokines, such as TNF-*α*, IL-1*β*, MCP-1, and Lp-PLA_2_ [11–13]. Thus, the present study was designed to elucidate the effect of SAA on the production of Visfatin.

The primary finding of the present study is that SAA stimulated the production of Visfatin. In cultured RAW264.7 macrophages, SAA time- and concentration-dependently upregulated the expression of Visfatin. From Figures 1(b) and 1(d), we could find that the Visfatin protein began to elevate after 12 h stimulation, while its mRNA elevated significantly after 6 h, which manifested the direct effect of SAA on the transcription and translation of Visfatin. Meanwhile, we also detected the concentration of Visfatin in the supernatants, which indicated that the stimulation of SAA also significantly promoted the secretion of Visfatin. Nevertheless, when the stimulation time reached 48 hours, the expression of Visfatin continued to increase, which reminded us that, except for the direct effect, there might be some indirect pathways involved in SAA-induced Visfatin production. As reported previously, SAA could stimulate the production of a series of cytokines in monocytes, such as TNF-*α*, IL-1*β*, IL-6, and IL-8 [11, 12, 19], while TNF-*α* has been considered as mediator of inflammation and mediated the expression of Visfatin [20]. In addition, primary monocytes were isolated and cultured, and the results of the primary monocytes also confirmed the effects of SAA on the production of Visfatin. Accordingly, we speculated that SAA might perform its durative effect on Visfatin production through these cytokines, which needs further investigation.

In addition, another important finding of the present study is that SAA induced the production of Visfatin through FPR2/ERK1/2/PPAR-*γ* signaling pathway. It has been reported that FPR2 is one of the most important receptors of SAA and mediated a variety of biological responses [10, 15, 19], so we then sought to clarify its influence on SAA-induced Visfatin expression. Using pharmacological agonist and antagonist, as well as FPR2 siRNA, we found that both WRW^4^ (an antagonist of FPR2) [21] and FPR2 siRNA could significantly abolish the promoting effect of SAA on expression of Visfatin, while WKYMVm (an agonist of FPR2) [22] could significantly promote the expression of Visfatin. The results above confirmed that SAA promoted the expression of Visfatin via FPR2.

Then, we detected the phosphorylation of ERK1/2, which has been seen as a downstream signaling molecule of SAA and played important roles in a series of biological processes that SAA participated in [17, 18]. We found that SAA could significantly induce phosphorylation of ERK1/2 after 15 min stimulation in cultured RAW264.7 macrophages, with the peak appearing at about 30 min after stimulation and then descending quickly after 60 min stimulation. Moreover, when we pretreated the cells with PD98059 (the specific inhibitors of ERK1/2), we found that the elevated expression of phosphoERK1/2 at 30 min after stimulation with SAA disappeared, which revealed that SAA induced Visfatin expression via the phosphorylation of ERK1/2. After that, we also detected the regulating effect of SAA on the nuclear transcriptional factor PPAR-*γ*, and the results demonstrated that SAA could significantly promote the expression of PPAR-*γ* after 3 h stimulation. Similar to the results of ERK1/2, the application of GW9662 (the specific antagonist of PPAR-*γ*) significantly inhibited the upregulating effect of SAA, while using Rosiglitazone (the specific agonist of PPAR-*γ*) alone could also significantly upregulate the expression of PPAR-*γ*. The results above suggested that SAA induced Visfatin expression via PPAR-*γ* pathway.

From the time points of the protein elevated, we could extrapolate that the activation sequence of the signaling molecules above might be FPR2/ERK1/2/PPAR-*γ*, because SAA-induced phosphorylation of ERK1/2 required 15–30 min, while SAA-induced elevation of PPAR-*γ* required no less than 3 h. In order to confirm the sequence of the signaling molecules, we blocked FPR2 with WRW^4^ and FPR2 siRNA, and both ERK1/2 and PPAR-*γ* were evidently inhibited, while activation of FPR2 with WKYMVm significantly activated ERK1/2 and PPAR-*γ*, which suggested that FPR2 was the upstream of ERK1/2 and PPAR-*γ* pathway. Then, we blocked the ERK1/2 pathway, and we also found that the expression of PPAR-*γ* was decreased, which confirmed that ERK1/2 was the upstream of PPAR-*γ* pathway.

Taken together, our study provides the evidence for the first time that SAA stimulates the production of Visfatin in RAW264.7 macrophages and primary monocytes. Furthermore, the second finding is that SAA induced Visfatin expression via FPR2/ERK1/2/PPAR-*γ* signaling pathway. According to our results, it reminded us again that SAA may be not only a prognostic indicator but also a proinflammatory mediator and SAA should be taken as a potential therapeutic target in the treatment of atherosclerosis.

## Supplementary Material

Supplemental Fig 1: SAA up-regulates Visfatin expression in cultured RAW264.7 cells. To further confirm the effect of SAA, we also purchased recombinant human apo-SAA1 from Sigma-Aldrich (St Louis, MO). RAW264.7 macrophages were again cultured and stimulated with SAA (50 µg/ml, Sigma, SRP4324) for 24 h. The expression of Visfatin was detected with western blot and real-time PCR respectively at protein level. ∗∗ *P* < 0.01 versus control group. Data shown are means ± SEM from three independent experiments in duplicate.
