# Supplementary material for: Serum Amyloid a Promotes Visfatin Expression in Macrophages
Source: Biomed Res Int. 2016 Feb 24;2016:4819327. doi: 10.1155/2016/4819327 (PMC4783535; doi:10.1155/2016/4819327)
Supplement: Supplementary file 1 — Supplemental Fig 1: SAA up-regulates Visfatin expression in cultured RAW264.7 cells. To further confirm the effect of SAA, we also purchased recombinant human apo-SAA1 from Sigma-Aldrich (St Louis, MO). RAW264.7 macrophages were again cultured and stimulated with SAA (50 µg/ml, Sigma, SRP4324) for 24 h. The expression of Visfatin was detected with western blot and real-time PCR respectively at protein level. ∗∗ P < 0.01 versus control group. Data shown are means ± SEM from three independent experiments in duplicate. [file 4819327.f1.doc]

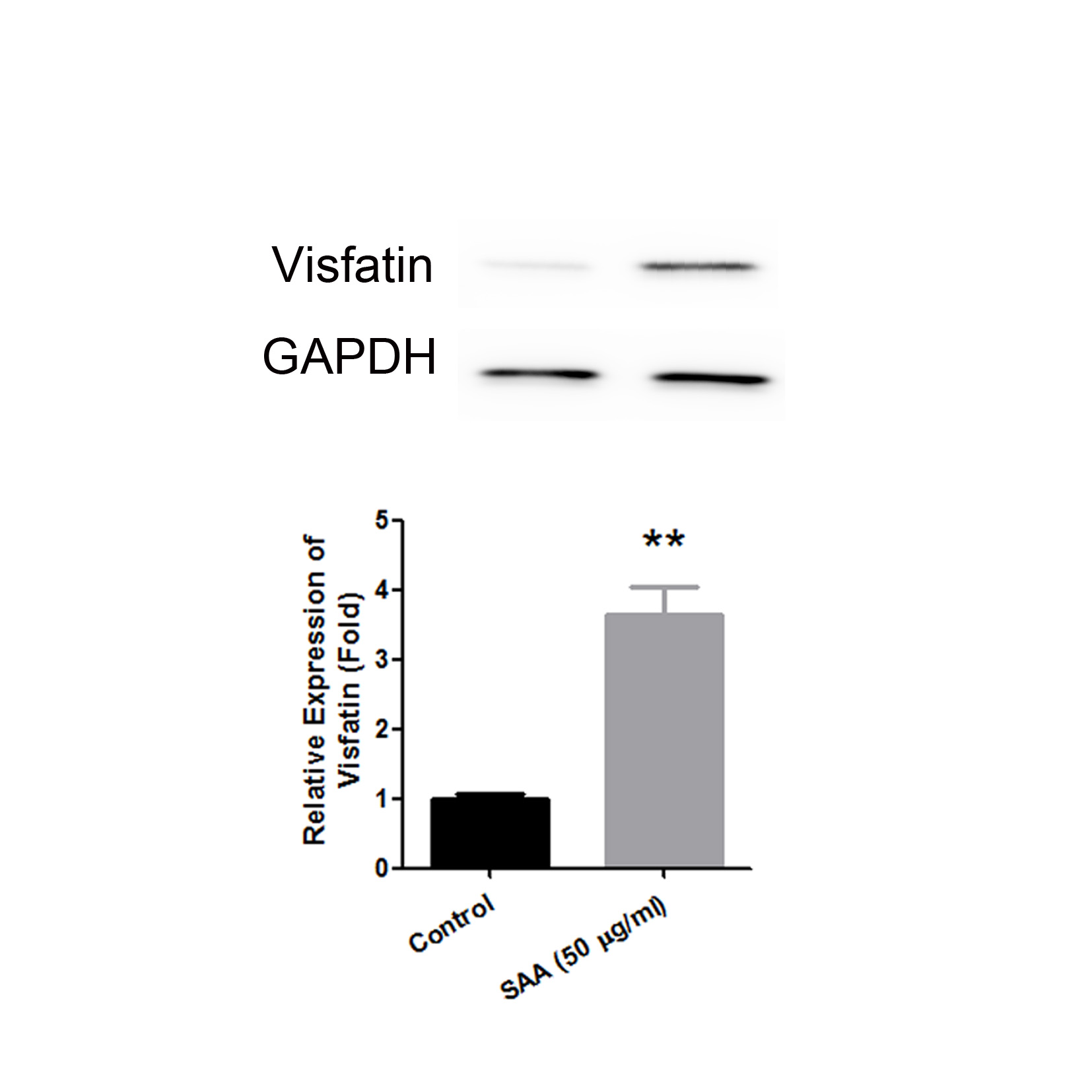


Supplemental Fig 1

RAW264.7 macrophages were cultured and stimulated with SAA (50 μg/ml, Sigma, [SRP4324](http://www.sigmaaldrich.com/catalog/product/sigma/srp4324)) for 24 h. The expression of Visfatin was detected with western blot at protein level. ** P<0.01 versus control group. Data shown are means ± SEM from three independent experiments in duplicate.
